# Menstrual Disorders in Nongenital Tuberculosis

**DOI:** 10.1155/IDOG/2006/18452

**Published:** 2006-02-02

**Authors:** Masoumeh Fallahian, Maryam Ilkhani

**Affiliations:** ^1^Infertility and Reproductive Health Research Centre, Taleghani Hospital, Shaheed Beheshti University of Medical Sciences and Health Services, Evin Avenue, Tehran 19395, Iran; ^2^Department of Gynecology and Obstetrics, School of Medicine, Shaheed Beheshti University of Medical Sciences and Health Services, Evin Avenue, Tehran 19395, Iran

## Abstract

Menstrual patterns differ even in nongenital tuberculosis. Our objective is to determine whether nongenital tuberculosis makes menstrual dysfunction, before and sustain after treatment. Menstrual patterns were compared in women with pulmonary or extrapulmonary but nongenital tuberculosis with healthy nursing students and also with themselves, before and after treatment in a retrospective cohort study. Subjects were selected by convenient nonrandomized sampling but control groups were selected by random allocation among volunteers of nursing students. Case and control subjects were matched in age group. Menstrual patterns including amount, duration, interval, cessation of period, any menstrual irregularity, and pelvic pain were evaluated. Among 100 cases of proven tuberculosis, 90 patients had pulmonary and 10 cases had extrapulmonary tuberculosis. Secondary amenorrhea (*P* ≤ .001, RR: 22), spotting during menstrual period (*P* ≤ .0001, RR: 4.5), decreasing in amount (*P* ≤ .001, RR: 7.8), shorter duration of menstrual period (*P* ≤ .001, RR: 12), and pelvic pain (*P* ≤ .001, RR: 8.6) were more prevalent and significantly different in the cases compared to control subjects (with CI:95% and *P* < .001), but excessive or prolong vaginal bleeding was not observed. Menstrual disorders occur even in nongenital tuberculosis, but it is manifested with cessation or decrease in menstrual bleeding flow and period.

## INTRODUCTION

Tuberculosis (TB) continues to be a major health problem in developing countries and compounded recently by dual infection of HIV and tuberculosis.

Genital tuberculosis is accompanied with menstrual disorders including hypomenorrhea, amenorrhea, and infertility. In one study, oligomenorrhea and amenorrhea were seen in 54% and 14% of genital tuberculosis, respectively [1], but estimation of menstrual dysfunctions in nongenital tuberculosis has been less studied. Active pulmonary tuberculosis without demonstrable lesions of structural involvement of either the ovaries or the genital tract is associated with amenorrhea and infertility in human females [2]. Hence, menstrual dysfunction is correlated with the severity of the disease being more common in severe forms of disease [3].

## METHOD

In a retrospective cohort study where exposure and outcome took place in the past period, menstrual patterns were compared in the patients with pulmonary or extrapulmonary but nongenital tuberculosis with healthy nursing students and with themselves, too; menstrual pattern including duration, interval, and amount of menstrual period, and also menstrual irregularity including excessive or prolong bleeding, decreasing in amount (hypomenorrhea), or retardation and cessation of menstrual period and also pelvic pain were compared before and after treatment. This study was conducted over an 11-month period in Masih Daneshvari Teaching Hospital affiliated to Shaheed Beheshti University of Medical Sciences and Health Services in Tehran where there is a referral center for treatment of TB. Case subjects were selected by convenient nonrandomized sampling but control groups were selected by random allocation among volunteers of nursing students. Case and control subjects were marched in age group. Case subjects were defined as the patients with proven tuberculosis by isolation of mycobacterium and positive culture of sputum or any material plus positive chest X-Ray in pulmonary cases of tuberculosis. Proven genital tuberculosis including pelvic mass, infertility with pelvic tuberculosis diagnosed by hysterosalpingography or positive menstrual and endometrial culture was excluded from this study. Control subjects were healthy nursing students that were checked for TB and any other communicable disease by taking of chest X-Ray, routine laboratory tests, and PPD at the setting of college admission. Approximately 100 persons of these students by random allocation accepted to participate and fill out the format questionnaire. If somebody refused to cooperate, another one was replaced.

Collected data were analyzed with SPSS software and *P*-value was considered meaningful at equal to or less than 0.05.

## RESULTS

Menstrual patterns were compared in one hundred cases and equal control subjects.

Approximately 90 cases had pulmonary tuberculosis and others had lymphatic tuberculosis in 8 cases, skeletal in one case, and peritoneal TB in one case.

Mean ages of the cases and controls were 27.5 ± 8.4 and 26.9 ± 5.5 years, respectively.

Menarche took place at the age of 13.77 ± 1.25 years in cases and 13.39 ± 1.3 years, in control subjects.

Secondary amenorrhea with a mean duration of 10.59 months took place in 22 cases of the patients but no one in control group. Therefore, menstrual disorders were compared in 78 cases with 100 control subjects. Some cases had hypomenorrhea together with shorter menstrual period ≤ 3 days. Menstrual disorders in cases and controls have been shown in Table 1.

Menstrual dysfunctions were compared in the patients, before and after a mean period of 7.1 months of treatment with anti-TB drugs including Isoniazide, Rifampine, Etambutol, or Streptomycin.

Comparison of menstrual disorders is shown in Table 2.

## COMMENT

TB is a leading cause of death among women of reproductive age and is estimated to cause more deaths among this group than all causes of maternal mortality [4].

Women are less likely than men to develop an infection, but are less likely to be tested and treated [5].

Hypomenorrhea and secondary amenorrhea widely seen in the patients with pulmonary tuberculosis may be resulting from dysfunction ascribed to hypothalamus, pituitary, and premature ovarian failure or even due to organic lesions in the uterine endometrium in one study [6]. Another study reveals that approximately 13% of women with pulmonary tuberculosis have genital tuberculosis and this result will be found by repeated histopathologic studies when clinical diagnosis is not possible [7]. Hypomenorrhea and secondary amenorrhea in the patients with extra genital tuberculosis with no involvement of ovary or genital tract are poorly understood which is in proportion to the severity of the disease.

There are some limitations in this study including undiagnosed genital tuberculosis among the cases and also socioeconomic differences between cases and controls, but these cases have been compared with themselves before and after treatment, too.

In endemic countries for tuberculosis including border areas of Iran, tuberculosis could be diagnosed when menstrual irregularity with hypomenorrhea and prolong interval periods are the whole TB manifestations. However, it needed to be proved in next studies. Timely and appropriate treatment restores normal menstrual function in some of these cases.

## Figures and Tables

**Table 1:** Comparison of menstrual patterns in the women, with (cases) and without tuberculosis (controls).

Menstrual pattern	Cases	Control	*P*-value	Relative risk
	no 78	no 100		

Secondary amenorrhea	22	0	≤ .001	22
Hypomenorrhea	26	6	≤ .001	7.8
Shorter menstrual period ≤ 3 days	34	6	≤ .001	12
Intermenstrual spotting	15	5	≤ .001	4.5
Menstrual irregularity	35	9	≤ .001	8.2
Longer menstrual period ≥ 8 days	2	8	≤ .001	0.328
Dysmenorrhea (mild to moderate)	52	75	≤ .001	0.66
Pelvic pain	44	13	≤ .001	8.6

**Table 2:** Menstrual patterns in the patients with tuberculosis, before and after treatment.

Menstrual pattern	After treatment	Before treatment	RR
	no 85	no 78	

Secondary amenorrhea	15	22	0.62
Intermenstrual spotting	13	15	0.75
Dysmenorrhea	51	52	0.75
Shorter menstrual period ≤ 3 days	30	34	0.7
Longer menstrual period ≥ 8 days	3	2	1.39
Menstrual irregularity	29	35	0.63
Pelvic pain	40	44	0.68
